# The association between early maladaptive schemas and glycaemic control in patients with type 2 diabetes mellitus: A cross‐sectional study

**DOI:** 10.1002/edm2.437

**Published:** 2023-07-04

**Authors:** Amin Sayyadi, Mohammad Mehdi Maleksaabet, Mohammad Hossein Gozashti

**Affiliations:** ^1^ Student Research Committee, School of Medicine Kerman University of Medical Sciences Kerman Iran; ^2^ Clinical Research Development Unit, Afzalipour Hospital Kerman University of Medical Sciences Kerman Iran; ^3^ Endocrinology and Metabolism Research Center Institute of Basic and Clinical Physiology Sciences, Kerman University of Medical Sciences Kerman Iran

**Keywords:** diabetic mellitus, early maladaptive schemas, mental health

## Abstract

**Introduction:**

Diabetes is a disease with high prevalence and causes heavy economic burden. Mental and physical health are tied together and their interaction determines one's health or sickness. Early maladaptive schemas (EMSs) are suitable indicators of mental health. We investigated the association between EMSs and glycaemic control in type 2 diabetes mellitus (T2DM) patients.

**Methods:**

We conducted a cross‐sectional study in 2021 on 150 patients with T2DM. We used two questionnaires a demographic data questionnaire, and a Young Schema Questionnaire 2 – Short Form for gathering the data. We also performed laboratory tests on our participants and used the results of fasting blood sugar and haemoglobin A_1_c to evaluate glycaemic control.

**Results:**

Most of our participants were females (66%). Most of our patients were 41–60 years old (54%). There were only three single participants, and 86.6% of our individuals did not have a university degree. Total mean ± SD for EMSs score was 192.45 ± 55.66; self‐sacrifice (19.09 ± 4.64) and defectiveness/shame (8.72 ± 4.45) had the highest and lowest EMSs scores, respectively. None of the demographic data had any significant impact on EMSs scores or glycaemic control, but generally, younger patients with higher levels of education had better glycaemic control. Participants with higher scores for defectiveness/shame and insufficient self‐control had significantly worse glycaemic control.

**Conclusion:**

Mental and physical health are tied together, and paying attention to psychological aspects in prevention and management of physical disorders is crucial. EMSs, especially defectiveness/shame and insufficient self‐control are associated with glycaemic control of T2DM patients.

## Graphical abstract

We conducted a cross‐sectional study to investigate the association between early maladaptive schemas, as suitable indicators of mental health, and glycaemic control in type 2 diabetes mellitus patients. Our results revealed that there are significant differences among patients with good and poor glycaemic control in terms of early maladaptive schemas, especially defectiveness/shame and insufficient self‐control. We are willing to encourage healthcare providers to consider mental aspects in the management of diabetic patients.

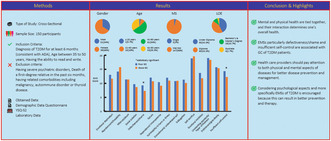

## 1 INTRODUCTION

Type 2 diabetes mellitus (T2DM) is a metabolic disorder characterized by hyperglycaemia due to peripheral insulin resistance and inadequate insulin secretion by pancreatic beta cells.^1^ Diabetes is a disease with high prevalence, and is still increasing: the latest report from the Centers for Disease Control and Prevention (CDC) revealed that 10.5% of the United States (US) population have diabetes and 34.5% of the population have prediabetes^2^; in 2021, the number of diabetic patients was 537 million people, International Diabetes Federation (IDF) has predicted that this number will rise to 783 million by 2045, especially in developing countries.^3^ Besides high prevalence, diabetes causes a heavy economic burden globally, it is projected that these consequences will rise substantially by 2030; Bommer et al. say that even if we meet international targets, we still cannot decrease the global economic burden.^4^ Diabetes complications are one of the substantial sources of its high economic burden: cardio‐cerebrovascular complications including coronaropathy and stroke; nephropathy; retinopathy, diabetic foot; etc.^5^ Iran is a country with a high prevalence of diabetes, prevalence is 8% in Iran and 9.6% in Kerman.^6^

Mental and physical health are tied together. Many scientists have shown this interaction between mental disorders and different diseases, including asthma,^7^ cardiovascular disease,^8^ autoimmune thyroiditis,^9^ etc. The same association has been found between diabetes and mental disorders, such as depression, mood disorder, and cognitive dysfunction.^5^, ^10^, ^11^

Early maladaptive schemas (EMSs) are some of the most crucial factors related to mental health.^12^ Young et al.^13^ defined it as ‘self‐defeating emotional and cognitive patterns that begin early in our development and repeat throughout life’, and named 15 EMSs in 5 domains: disconnection & Rejection, impaired autonomy & performance, impaired Limits, other‐directedness, over‐vigilance & inhibition.

The interaction between mental factors and physical health is clear, and studies regarding the interaction between EMS and glycaemic control are few. Herein, we conducted a cross‐sectional study to investigate the association of EMSs with glycaemic control in T2DM patients.

## 2 METHODS

### 2.1 Participants and procedures

In 2021, we performed a cross‐sectional study to investigate the association between EMSs with glycaemic control in patients with T2DM at Afzalipour Hospital and a related clinic, in Kerman, Iran.

Inclusion criteria were diagnosis of T2DM for at least 6 months (consistent with American Diabetes Association^14^), age between 35 and 50 years, and having the ability to read and write.

Exclusion criteria were having severe psychiatric disorders, the death of a first‐degree relative in the past 6 months, and having related comorbidities including malignancy, autoimmune disorder or thyroid disease.

### 2.2 Measures

After explaining the study and obtaining informed consent from the participants, an internal medicine resident used two questionnaires to gather data: a demographic data questionnaire, and a Young Schema Questionnaire 2 – Short Form (YSQ‐S2).

Demographic data consisted of gender, age, and level of education. YSQ‐S2 has 75 five‐point Likert scale questions on different aspects of 15 EMSs in five categories: disconnection/rejection (abandonment/instability, mistrust/abuse, emotional deprivation, defectiveness/shame, social isolation), impaired autonomy/performance (dependence/incompetence, failure, vulnerability to harm or illness, enmeshment/undeveloped self), impaired limits (entitlement/grandiosity, insufficient self‐control), other directedness (subjugation, self‐sacrifice), and over vigilance/inhibition (emotional inhibition, unrelenting standards/hyper criticalness). The reliability and validity of this questionnaire have been certified before.^15^ Since we conducted this study in the Persian population, we used the Persian translation of YSQ‐S2, which was provided and verified by Khosravani and colleagues.^16^ They reported that the reliability and validity of this questionnaire are suitable (Cronbach's alpha; total: 0.76, Emotional deprivation: 0.87, Abandonment/instability: 0.76, Mistrust/abuse: 0.71, Social isolation; 0.76, Defectiveness/shame: 0.79, Failure: 0.80, Dependence/incompetence: 0.74, Vulnerability to harm or illness: 0.76, Enmeshment/undeveloped self: 0.78, Subjugation: 0.73, Self‐sacrifice: 0.70, Emotional Inhibition: 0.73, Unrelenting standards/hyper criticalness: 0.72, Entitlement/grandiosity: 0.72, Insufficient self‐control: 0.71).

We also evaluated patients' laboratory results including fasting blood sugar (FBS), haemoglobin A_1_c (HbA_1_c), triglyceride (TG), low‐density lipoproteins (LDL), high‐density lipoproteins (HDL) and cholesterol (Chol). Based on the criteria provided by ADA,^17^, ^18^ FBS of 80–130 mg/dL (4.4–7.2 mmol/L), HbA_1_c of <7%, TG < 150 mg/dL (1.7 mmol/L), HDL ≥ 40 mg/dL [1.0 mmol/L] for men and ≥ 50 mg/dL (1.3 mmol/L) for women were considered suitable.

### 2.3 Statistical analysis

We used measures of central tendency and dispersion for descriptive analysis and Independent *T*‐test, ANOVA, and chi‐square for inferential analysis. We have presented the data in tables and charts. SPSS version 24 manufactured by IBM was our statistical analysis software. p‐value <.05 was considered statistically significant.

### 2.4 Ethical considerations

This project was reviewed and approved by the ethics committee of the Kerman University of Medical Sciences with the licence number IR.KMU.AH.REC.1401.046. Also, informed consent was obtained from all the participants.

## 3 RESULTS

**FIGURE 1:**
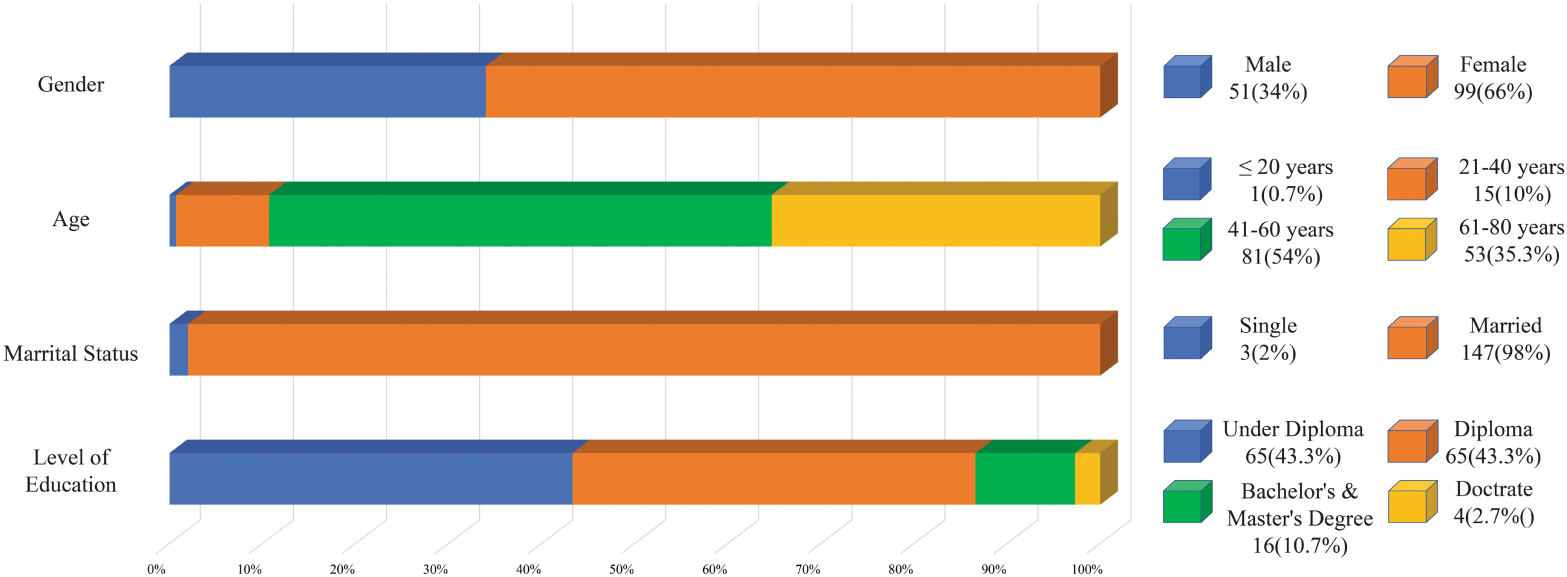
Demographic data of the participants.

Our population consisted of 150 individuals (66% females). More than half of our patients were middle‐aged (54%), and young patients comprised only 10.7% of our participants. There were only three single patients in our population. Having a university degree was not a common trait (Diploma and under Diploma = 86.6%) (Figure 1).

The total mean ± SD for EMSs was 192.45 ± 55.66. The highest and lowest scores were for self‐sacrifice (19.09 ± 4.64) and defectiveness/shame (8.72 ± 4.45), respectively (Table 1).

**TABLE 1:** Descriptive and inferential statistics of early maladaptive schemas and their related glycaemic control.

EMSs	EMSs scores	Poor glycaemic control	Good glycaemic control	*p*‐value (glycaemic control)
	Mean ± SD	Mean ± SD	Mean ± SD	
Emotional deprivation	12.64 ± 6.18	13 ± 0.6	11.68 ± 0.91	.24
Abandonment/instability	14.62 ± 6.05	14.19 ± 0.59	15.75 ± 0.88	.15
Mistrust/abuse	11.36 ± 5.1	11.36 ± 0.47	11.34 ± 0.85	.97
Social isolation	9.42 ± 4.9	9.7 ± 0.47	8.65 ± 0.75	.24
Defectiveness/shame	8.72 ± 4.45	9.26 ± 0.46	7.29 ± 0.42	.002
Failure	11.68 ± 5.24	12.03 ± 0.51	10.73 ± 0.74	.17
Dependence/incompetence	10.05 ± 5.19	10.4 ± 0.5	9.12 ± 0.78	.17
Vulnerability to harm or illness	11.7 ± 6.01	11.98 ± 0.58	10.95 ± 0.91	.35
Enmeshment/undeveloped self	10.82 ± 4.79	10.79 ± 0.47	10.9 ± 0.67	.9
Subjugation	10.94 ± 5.44	11.25 ± 0.53	10.09 ± 0.76	.24
Self‐sacrifice	19.09 ± 4.64	18.88 ± 0.46	19.63 ± 0.64	.38
Emotional inhibition	13.18 ± 6.13	13.69 ± 0.59	11.82 ± 0.89	.09
Unrelenting standards/hyper criticalness	18.82 ± 4.57	19 ± 0.43	18.34 ± 0.72	.43
Entitlement/grandiosity	15.63 ± 5.29	15.72 ± 0.5	15.39 ± 0.82	.73
Insufficient self‐control	13.75 ± 6.11	14.32 ± 0.61	12.24 ± 0.79	.04
Total	192.45 ± 55.66	195.64 ± 5.58	183.97 ± 7.43	.25

Evaluation of laboratory results showed that the mean ± SD of FBS (160.12 ± 68.12 mg/dL) and HbA_1_C (8.09 ± 1.86%) in our population were higher than the target FBS for T2DM patients (130 mg/dL, 7%). The mean ± SD for TG was 163.65 ± 86.36 mg/dL which is higher than the target range of <150 mg/dL. The mean for LDL (79.68 ± 33.66 mg/dL) was in the optimal range (<100 mg/dL). The amount of HDL (46.17 ± 11.23 mg/dL) was suitable when considering the optimal range for men (>40 mg/dL), but it was not ideal if we consider the optimal range for women (>50 mg/dL). The mean ± SD of Chol was 152.96 ± 42.18 mg/dL.

Assessment of FBS and HbA_1_C revealed that the majority of our patients had good glycaemic control (109 participants, 72.7%).

The mean ± SD of EMSs in women was higher than in men (197.35 ± 56.6 vs. 182.94 ± 53.05). The age category with the highest and lowest means were ≤ 20 years and 21–40 years, respectively. Single patients had higher EMSs scores than those who were married (224 ± 33.65 vs. 191.8 ± 4.58). The highest and the lowest EMSs scores were for the patients with an under‐Diploma degree and Doctorate, respectively: the higher the education level, the less the score of EMSs. None of the demographic variables had any significant impact on EMSs scores (Table 2).

**TABLE 2:** Descriptive and inferential statistics of participants' early maladaptive schemas scores and glycaemic control.

Variable	EMSs scores	*p*‐value (EMSs scores)	Good glycaemic control	Poor glycaemic control	*p*‐value (glycaemic control)
	Mean ± SD		Frequency(%)	Frequency(%)	
Gender					
Male	182.94 ± 53.05	0.13	41(80.4)	10(19.6)	.12
Female	197.35 ± 56.6		68(68.7)	31(31.3)	
Age					
≤20 years	263	0.64	1(100)	0	.48
21–40 years	188.86 ± 16.6		13(86.7)	2(13.3)	
41–60 years	191.91 ± 6		59(72.8)	22(27.2)	
61–80 years	192.96 ± 7.79		36(68)	17(32)	
Marital status					
Single	224 ± 33.65	0.32	2(66.7)	1(33.3)	.81
Married	191.8 ± 4.58		107(72.8)	40(27.2)	
Level of education					
Under Diploma	198.83 ± 7.08	0.30	42(64.6)	23(35.4)	.19
Diploma	192.04 ± 7.09		51(78.5)	14(21.5)	
Bachelor's and Master's degree	176.93 ± 11.03		12(75)	4(25)	
Doctorate	157.5 ± 15.98		4(100)	0	

No demographic variable had any significant effect on glycaemic control (*p*‐value > .05). Both men and women had high ratios of good glycaemic control, but the ratio was higher in men (80.4% vs. 68.7%). Although all of the age groups had high ratios of good glycaemic control, the ratio was higher in younger patients. When evaluating the association of glycaemic control with a level of education, the overall trend was that people with higher levels of education had better glycaemic control, but individuals with Diploma had a slightly higher ratio of good glycaemic control than the group with Bachelor's & Master's degree. All of the four patients with Doctorate had good glycaemic control (Table 2).

The total mean ± SD of the EMSs for the patients, who had poor glycaemic control (195.64 ± 5.58) was higher than the group with good glycaemic control (183.97 ± 7.43). All of the EMSs' mean ± SD were higher in the group with poor glycaemic control except for abandonment/instability, enmeshment/undeveloped self, and Self‐sacrifice. Only two EMSs scores had significant differences (*p*‐value <.05) between the two groups: defectiveness/shame and insufficient self‐control (Table 1).

## 4 DISCUSSION

We conducted a cross‐sectional study to investigate the association between EMSs and glycaemic control in patients with T2DM. We had a high ratio of good glycaemic control in our population. No demographic variable had any significant impact on glycaemic control, but younger patients and those who had higher levels of education had better glycaemic control. Patients with poor glycaemic control had higher EMSs scores, and the differences in defectiveness/shame and insufficient self‐control were significant.

Fathabadi et al.^19^ worked on predicting the blood glucose levels in T2DM patients based on the EMSs, they found that social isolation/alienation, defect/shame, vulnerability to disease, obedience, emotional inhibition, endurance/over‐critical criteria, and inadequate self‐discipline/self‐discipline were significantly related to poor blood glucose levels. They concluded that EMSs can predict blood glucose levels in T2DM patients and suggested paying attention to this aspect in preventive and therapeutic interventions.

Mirdrikvand et al.^20^ compared EMSs between patients with and without T2DM; they found that abandonment, failure, vulnerability, enmeshment, self‐sacrifice, entitlement, and insufficient self‐control schemas as well as the over‐vigilance and inhibition schematic domains are higher in diabetic patients.

We had a high ratio of good glycaemic control in our study (72.7%). Gebreyohannes et al.^21^ did a systematic review and meta‐analysis on glycaemic control in type 1 and 2 diabetic patients; they found 34.4% and 33.2% glycaemic control based on FBS and HbA_1_C measurements. Pérez‐Losada et al.^22^ set the threshold of good glycaemic control at 6.5% for HbA_1_C; they reported that 21.8% of T2DM patients had good glycaemic control. Yigazu and Desse^23^ found that 40.8% of their patients had good glycaemic control based on the level of FBS. In our study most of the participants were clinic patients who had good insight into their condition, good cooperation, and regular visits to the physician; these could be the potential reasons for a higher ratio of glycaemic control. Differences in the level of education, socioeconomic status, and not using the same and standardized methods of measurement are the other possible reasons for this disparity in the literature.

None of the demographic variables had any significant effect on glycaemic control, but numbers show that patients with higher levels of education had higher ratios of glycaemic control: 12 out of 16 patients with Bachelor's and Master's degrees had glycaemic control, all of the 4 patients with Doctorate had good glycaemic control. Multiple studies support the idea that a higher level of education leads to a higher ratio of glycaemic control.^24^, ^25^, ^26^, ^27^

Although all our age categories had high ratios of good glycaemic control, younger patients had the highest ratio of glycaemic control. This is in contrast with other studies that have shown that older people have better glycaemic control.^28^, ^29^, ^30^ These studies emphasize the idea that because older patients are more prone to complications of diabetes, these potential consequences make them more responsible for following their treatment. The other reason that these studies mention is that younger patients have more unhealthy diets. We believe that some factors are neglected: younger patients are more skilful in controlling their blood sugar by using different gadgets and medications; modern medications including Sodium‐Glucose Cotransporter‐2 (SGLT2) Inhibitors and Dipeptidyl Peptidase‐4 (DPP‐4) Inhibitors are more effective in glycaemic control, and using non‐insulin medications is more common among younger patients; using insulin is more common among older patients and glycaemic control is usually less achievable in insulin users.

The total mean of EMSs was higher in patients with poor glycaemic control (195.64 vs. 183.97), but it was not statistically significant. The only significant impacts were defectiveness/shame and insufficient self‐control.

Defectiveness/shame comes from the belief of not being fundamentally worthy. This schema causes individuals to be oversensitive to criticism, rejection, and blame. This schema also includes the feeling of being despicable and worthless, which can explain the significant connection between defectiveness/shame and having poor glycaemic control, because these people do not know themselves as worthy of proper care and treatment.^15^, ^19^

Insufficient self‐control is a schema, in which an individual has difficulty in self‐control and distress tolerance. They are also more prone to be defeated by temptations and impulses. Achieving optimal glycaemic control demands a high level of self‐control and the ability to resist temptations; based on these explanations, we realize that individuals with insufficient self‐control are more prone to not having good glycaemic control.^31^

## 5 LIMITATIONS

We conducted this study in a clinic with a patient population that has regular visits to a physician, this can cause bias because there are many patients with improper care and irregular visits to a physician. Also, many patients were not willing to participate, so we had to eliminate them from the study; this is another possible root of bias.

We suggest doing studies with more population. Doing the study with a more diverse population and in different settings (inpatient and outpatient) is also recommended.

## 6 CONCLUSION

Mental and physical health are two inseparable components, and their interaction determines one's health. We believe that this study can show that EMSs, as a good indicator of mental health, are well associated with glycaemic control. Considering psychological aspects and more specifically EMSs is encouraged because this can result in better prevention and therapy of T2DM.

## AUTHOR CONTRIBUTIONS

**Amin Sayyadi:** Formal analysis (equal); software (lead); visualization (lead); writing – original draft (lead); writing – review and editing (equal). **Mohammad Mehdi Maleksaabet:** Data curation (lead); formal analysis (equal); investigation (equal). **Mohammad Hossein Gozashti:** Methodology (lead); project administration (equal); supervision (lead); writing – review and editing (equal).

## FUNDING INFORMATION

This research did not receive any specific grant from funding agencies in the public, commercial, or not‐for‐profit sectors.

## CONFLICT OF INTEREST STATEMENT

The authors have no conflict of interest.

## DISCLOSURE

The authors declare that there are no conflicts of interest regarding the publication of this paper.

## ETHICS STATEMENT

Ethics approval and consent to participate Informed consent was received from the patient before starting the work and the study was approved by the ethics committee of Kerman University of Medical Sciences (Code: IR.KMU.AH.REC.1401.046).

## PATIENT CONSENT STATEMENT

Written consent was obtained from the patient regarding publishing this case report in accordance with the journal's patient consent policy.

## Data Availability

we have put the available data in this article, but in case of any questions you can directly contact the corresponding author.

## References

[1] Hussain A , Claussen B , Ramachandran A , Williams R . Prevention of type 2 diabetes: a review. Diabetes Res Clin Pract. 2007;76:317‐326. 17069920 10.1016/j.diabres.2006.09.020

[2] National Diabetes Statistics Report 2020 in, Centers for Disease Control and Prevention, National Diabetes Statistics Report website.

[3] IDF Diabetes Atlas, in: Idf diabetes atlas, International Diabetes Federation © International Diabetes Federation, 2021, Brussels, 2021.

[4] Bommer C , Sagalova V , Heesemann E , et al. Global economic burden of Diabetes in adults: projections from 2015 to 2030. Diabetes Care. 2018;41:963‐970. 29475843 10.2337/dc17-1962

[5] Schlienger JL . Type 2 diabetes complications. Presse Med. 2013;42:839‐848. 23528336 10.1016/j.lpm.2013.02.313

[6] Najafipour H , Sanjari M , Shokoohi M , et al. Epidemiology of diabetes mellitus, pre‐diabetes, undiagnosed and uncontrolled diabetes and its predictors in general population aged 15 to 75 years: a community‐based study (KERCADRS) in southeastern Iran. J Diabetes. 2015;7:613‐621. 25042896 10.1111/1753-0407.12195

[7] Hayatbakhsh MR , Najman JM , Clavarino A , Bor W , Williams GM , O’Callaghan MJ . Association of psychiatric disorders, asthma and lung function in early adulthood. J Asthma. 2010;47:786‐791. 20690799 10.3109/02770903.2010.489141

[8] Mathews L , Ogunmoroti O , Nasir K , et al. Psychological factors and their association with ideal cardiovascular health among women and men. J Womens Health (Larchmt). 2018;27:709‐715. 29377738 10.1089/jwh.2017.6563 PMC5962331

[9] Mizokami T , Wu Li A , El‐Kaissi S , Wall JR . Stress and thyroid autoimmunity. Thyroid. 2004;14:1047‐1055. 15650357 10.1089/thy.2004.14.1047

[10] McCrimmon RJ , Ryan CM , Frier BM . Diabetes and cognitive dysfunction. Lancet. 2012;379:2291‐2299. 22683129 10.1016/S0140-6736(12)60360-2

[11] Bădescu SV , Tătaru C , Kobylinska L , et al. The association between Diabetes mellitus and depression. J Med Life. 2016;9:120‐125. 27453739 PMC4863499

[12] Bidadian M , Bahramizadeh H , Poursharifi H . Obesity and quality of life: the role of early maladaptive schemas. Procedia Soc Behav Sci. 2011;30:993‐998.

[13] Young JE , Klosko JS , Weishaar ME . Schema Therapy: A practitioner's Guide. Guilford Press; 2006.

[14] A. Association . 2. Classification and diagnosis of Diabetes: standards of medical Care in Diabetes–2021. Diabetes Care. 2021;44:S15‐S33. 33298413 10.2337/dc21-S002

[15] Abbasgholi ZGM , Khosravi Z , Ameri F . Comparison of Early Maladaptive Schemas and their Parental Origins and Coping Strategies in Psoriasis Patients, Diabetes Type 1 Patients and Healthy People. 2015.

[16] Khosravani V , Najafi M , Mohammadzadeh A . The Young schema questionnaire‐short form: a Persian version among a large sample of psychiatric patients. International Journal of Mental Health and Addiction. 2020;18:949‐967.

[17] American Diabetes A . Standards of medical Care in Diabetes—2022 abridged for primary care providers. Clinical Diabetes. 2022;40:10‐38. 35221470 10.2337/cd22-as01 PMC8865785

[18] A.D. Association . Dyslipidemia Management in Adults with Diabetes. Diabetes Care. 2004;27:s68‐s71. 14693930 10.2337/diacare.27.2007.s68

[19] Fathabadi J , Doulabi MHG , Arjmandnia AA , Adibi H , Shalani B , Sadeghi S . Prediction of blood glucose level in patients with type 2 diabetes through early maladaptive schemas. Daneshvar Medicine. 2020;28:40‐49.

[20] Mirdrikvand F , Sepahvandi MA , Khodarahimi S , Gholamrezaei S , Rahimian Bougar M , Shafikhani P . Early maladjustment schemas in individuals with and without type 2 Diabetes mellitus. Journal of Mind and Medical Sciences. 2019;6:150‐156.

[21] Gebreyohannes EA , Netere AK , Belachew SA . Glycemic control among diabetic patients in Ethiopia: a systematic review and meta‐analysis. PLoS One. 2019;14:e0221790. 31454396 10.1371/journal.pone.0221790 PMC6711596

[22] Pérez‐Losada FL , López‐López J , Martín‐González J , Jané‐Salas E , Segura‐Egea JJ , Estrugo‐Devesa A . Apical periodontitis and glycemic control in type 2 diabetic patients: cross‐sectional study. J Clin Exp Dent. 2020;12:e964‐e971. 33154799 10.4317/jced.57191 PMC7600214

[23] Yigazu DM , Desse TA . Glycemic control and associated factors among type 2 diabetic patients at Shanan gibe hospital. Southwest Ethiopia, BMC Res Notes. 2017;10:597. 29141693 10.1186/s13104-017-2924-y PMC5688756

[24] Mizokami‐Stout K , Choi H , Richardson CR , Piatt G , Heisler M . Diabetes distress and glycemic control in type 2 Diabetes: mediator and moderator analysis of a peer support intervention. JMIR Diabetes. 2021;6:e21400. 33427667 10.2196/21400 PMC7834928

[25] Bukhsh A , Khan TM , Sarfraz Nawaz M , Sajjad Ahmed H , Chan KG , Goh BH . Association of diabetes knowledge with glycemic control and self‐care practices among Pakistani people with type 2 diabetes mellitus. Diabetes Metab Syndr Obes. 2019;12:1409‐1417. 31616171 10.2147/DMSO.S209711 PMC6698595

[26] Reynolds DB , Walker RJ , Campbell JA , Egede LE . Differential effect of race, education, gender, and language discrimination on glycemic control in adults with type 2 diabetes. Diabetes Technol Ther. 2015;17:243‐247. 25549154 10.1089/dia.2014.0285 PMC4365429

[27] Hu J , Kline DM , Tan A , et al. Association between social determinants of health and glycemic control among African American people with type 2 diabetes: the Jackson heart study. Ann Behav Med. 2022;56:1300‐1311. 36197118 10.1093/abm/kaac026 PMC9672347

[28] Shamshirgaran SM , Mamaghanian A , Aliasgarzadeh A , Aiminisani N , Iranparvar‐Alamdari M , Ataie J . Age differences in diabetes‐related complications and glycemic control. BMC Endocr Disord. 2017;17:25. 28472985 10.1186/s12902-017-0175-5 PMC5418847

[29] Naranjo DM , Jacobs EA , Fisher L , Hessler D , Fernandez A . Age and glycemic control among low‐income Latinos. J Immigr Minor Health. 2013;15:898‐902. 22843322 10.1007/s10903-012-9689-0

[30] Comellas M , Marrero Y , George F , Matthews L . Age and glycemic control among adults with type 2 diabetes in the United States: an assessment from the National Health and nutrition examination survey (NHANES) 2013‐2014. Diabetes Metab Syndr. 2019;13:3069‐3073. 31765980 10.1016/j.dsx.2019.11.004

[31] Basile B , Tenore K , Mancini F . Early maladaptive schemas in overweight and obesity: a schema mode model. Heliyon. 2019;5:e02361. 31687536 10.1016/j.heliyon.2019.e02361 PMC6819863

